# Towards hippocampal navigation for brain–computer interfaces

**DOI:** 10.1038/s41598-023-40282-7

**Published:** 2023-08-28

**Authors:** Jeremy Saal, Maarten Christiaan Ottenhoff, Pieter L. Kubben, Albert J. Colon, Sophocles Goulis, Johannes P. van Dijk, Dean J. Krusienski, Christian Herff

**Affiliations:** 1 Maastricht University, Universiteitssingel 50, 6299 ER Maastricht, The Netherlands; 2 University of California, San Francisco, 675 Nelson Rising Ln, San Francisco, CA 94158 USA; 3 Academic Center for Epileptology Kempenhaeghe/MUMC, Kempenhaeghe, Heeze, The Netherlands; 4 Virginia Commonwealth University, Richmond, VA USA

**Keywords:** Neural decoding, Navigation

## Abstract

Automatic wheelchairs directly controlled by brain activity could provide autonomy to severely paralyzed individuals. Current approaches mostly rely on non-invasive measures of brain activity and translate individual commands into wheelchair movements. For example, an imagined movement of the right hand would steer the wheelchair to the right. No research has investigated decoding higher-order cognitive processes to accomplish wheelchair control. We envision an invasive neural prosthetic that could provide input for wheelchair control by decoding navigational intent from hippocampal signals. Navigation has been extensively investigated in hippocampal recordings, but not for the development of neural prostheses. Here we show that it is possible to train a decoder to classify virtual-movement speeds from hippocampal signals recorded during a virtual-navigation task. These results represent the first step toward exploring the feasibility of an invasive hippocampal BCI for wheelchair control.

## Introduction

Millions of people suffer from paralysis: The inability to move some part of their body^1^. In the most severe forms of paralysis, e.g. quadriplegia, individuals experience loss of control of their arms, legs, and torso. Paralysis can result from several diseases such as spinal cord injuries, transverse myelitis, multiple sclerosis, polio, and amyotrophic lateral sclerosis (ALS). These patients require assistance from family members and healthcare professionals. As the ability to independently interact with the environment is positively associated with life satisfaction^2^, it is imperative to develop solutions to provide independence to those with severe paralysis.

Brain–computer Interfaces (BCIs) allow communication between humans and computers without the need for muscle movement by decoding neural signals^3^. In recent years, researchers have demonstrated the potential of BCI to help several patient groups^4^. A study surveyed individuals with ALS and found that robotic arm and wheelchair control was of the highest priority when it comes to BCI development^5^. There has been great progress toward the development of robotic arm prostheses^6^. Wheelchair control, on the other hand, has received less attention from researchers, especially using invasive measures of brain activity.

Non-invasive BCI approaches such as those using electroencephalography (EEG), benefit from being low-risk as they record brain activity from outside of the scalp. However, in doing so, these approaches greatly compromise signal quality, spatial resolution, and/or temporal resolution^7^. Due to these limitations, EEG-based BCI for wheelchair control has not made major progress in recent years.

Many approaches using EEG directly or indirectly depend on eye movement and blinking^8–10^, translating them into simple directional commands. Eye movement and blinking are not feasible for many ALS patients^4^. Further, limiting eye movement during navigation would be inconvenient and hinder social interactions. Other methods rely on decoding mental tasks or imagery to indicate the intended movement^11–14^. For example, imagining mental rotation or left-hand movement to turn left. While using this method can provide high accuracy, the rate at which they transfer information is too slow for safe wheelchair use. An invasive BCI leveraging goal-oriented navigational intent may provide the input necessary for precise, safe, and intuitive wheelchair control. Brain–computer interfaces that decode higher-order cognitive processes may provide people with paralysis with an intuitive input for controlling external devices, thus promoting independence.

The higher signal-to-noise ratio, temporal resolution, and spatial accuracy of invasively recorded signals may allow for the development of improved wheelchair control. So far, all such research we are aware of has been conducted with non-human primates. Some studies have used joystick-based BCI paradigms for wheelchair control^15,16^. Hand movements were decoded from primary motor cortex neurons while the monkey used a joystick to control a wheelchair. Rajangam et al.^17^, on the other hand, showed the capability of rhesus monkeys to control a wheelchair based on invasive neural signals without using a joystick paradigm. This is an important step as many paralyzed individuals won’t be able to use a joystick to train the BCI classifiers. Using ensemble recordings in premotor and sensorimotor areas, the monkeys were able to control the rotational and translational movement of a wheelchair to reach their goal. This approach is promising as it was based on populations of neurons that were tuned for whole-body displacement. However, this approach decoded individual movement commands issued continuously, instead of decoding high-level planned trajectories.

BCIs based on the decoding of higher-level cognitive processes may provide intuitive and flexible BCI control by not requiring continuous input of movement commands. Decoding cognitive processes face many challenges, but researchers have had success in decoding decision-related processes (see^18^). Further, Mussalam et al.^19^ decoded higher-level goal representations from regions associated with reaching using electrode arrays. Of particular relevance, researchers have had great success in decoding navigation-related processes from the rodent hippocampus. Brown et al. used neural population activity to decode an accurate prediction of a rodent’s two-dimensional position within its environment^20^. Agarwal et al.^21^ extended this finding by showing that self-location can be decoded using both spiking and LFP activity. Further, researchers have had success decoding planned trajectories^22^.

While there is a wealth of research demonstrating the decoding of positional and navigational information from the hippocampus in rodents, few studies have explored what can be decoded from the human hippocampus. It has been shown that low-frequency oscillations power increases with movement speed in a virtual environment^23^. Vass et al.^24^ reported successful decoding of teleportation distance during a virtual-navigation task. Another study used microelectrode recordings to decode navigational goal information from spike phases from medial-temporal lobe structures, including the hippocampus^25^. Another study used a neural network to decode real-world movement speed from the hippocampus. Using a rare patient group with wireless intracortical electrodes implanted in the hippocampus, Aghajan et al. were able to record real-world ambulatory movement whilst recording neural activity^26^. They tracked participants’ speed as they were instructed to either walk at a slow or fast rate. They used a neural network to predict the top and slowest 30% of movement speeds based on spectral data.

**Figure 1:**
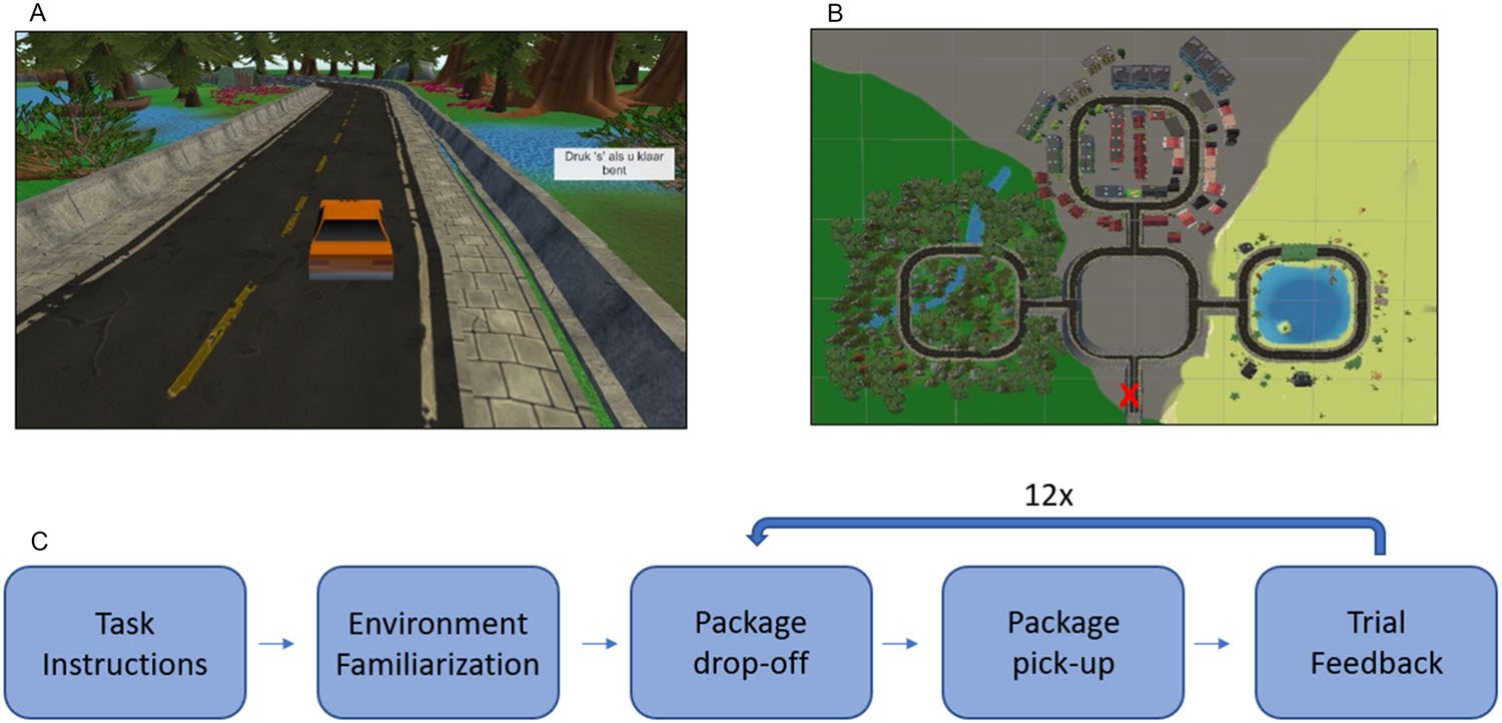
Virtual Navigation Task. (**a**) Participant’s view while navigating through the forest environment; (**b**) Top down view of the environment; presented to participants at the beginning. The starting position is indicated by the red X; (**c**) Schematic showing task structure.

Further research is needed to determine which navigational features can be decoded from human hippocampal activity. In the present study, we assess the extent to which virtual-movement speed can be decoded from invasively recorded hippocampal activity. Hippocampal activity was recorded during a keyboard-controlled virtual-navigation task in three patients. In the main portion of this task, participants navigated a car to three different locations (beach, forest, city) to drop off a package, which they had to retrieve again in the following trial (Fig. 1). Decoders were then trained to classify virtual speed from the theta and gamma activity. We show that, for all three patients, a classifier can discriminate between slow and fast virtual-movement speeds. Further, we show a classifier can discriminate between finer-grained speed levels.

In addition to our focus on decoding hippocampal activity for potential applications in wheelchair control for paralyzed individuals, we acknowledge the importance of exploring other methods for asynchronous, real-time control of virtual game objects, robotic arms, and other assistive devices. There are several invasive and non-invasive studies that have demonstrated the feasibility of such control (see^4^). Our study aims to contribute to this growing body of literature by examining the potential of hippocampal activity for BCI control in a virtual navigation context, laying the groundwork for future research in developing more intuitive and effective BCI solutions.

## Results

### Decoding slow vs. fast virtual-movement speed

**Figure 2:**
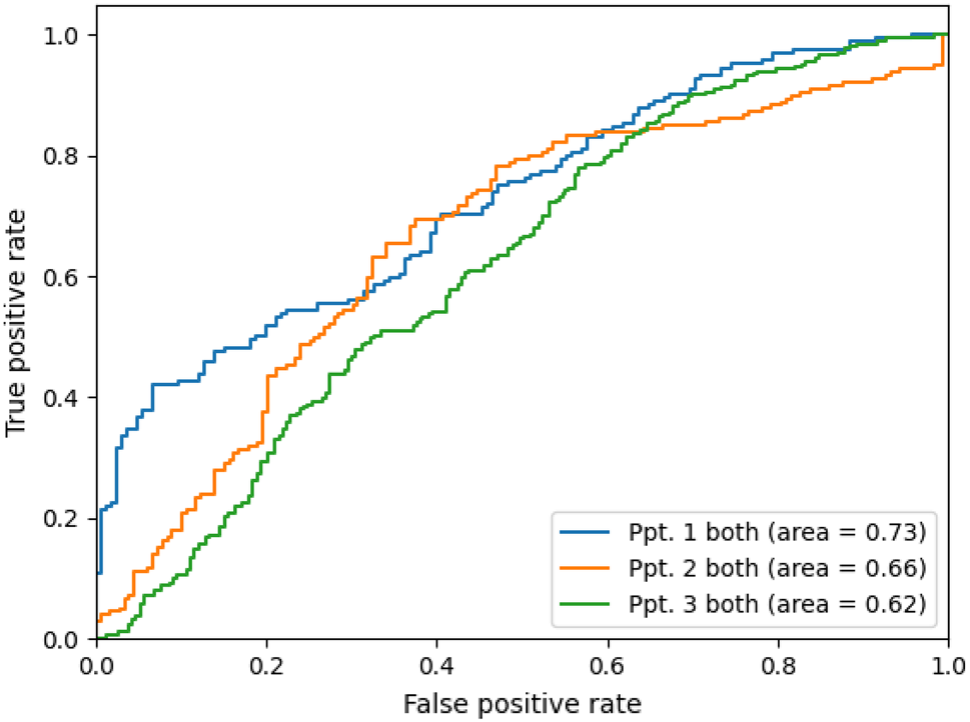
ROC curve for top vs bottom 10% of speeds. ROC plot showing classification of top vs bottom 10% of speeds. The area under the curve (AUC) for each patient is reported in the bottom right.

Theta and gamma power from hippocampal contacts were utilized as features in all decoding methods. Using a linear discriminant analysis (LDA), we were able to classify the fastest and slowest 10% of speeds above chance level for all participants (Fig. 2). For all patients, an equal number of high-speed and low-speed samples were used in classification. For P01, there were 164 samples each for slow and fast speeds; for P02, there were 179 samples each for slow and fast speeds; and for P03, there were 263 samples each for slow and fast speeds. A bootstrap resampling method was used to determine significant area under the curve (AUC) value (see “Methods” section). For all three participants, AUC values were significantly above chance (*p* < 0.05) when using theta and gamma features combined with AUC of 0.72, 0.66, and 0.62 respectively.

### Classifying broad speed groups

**Figure 3:**
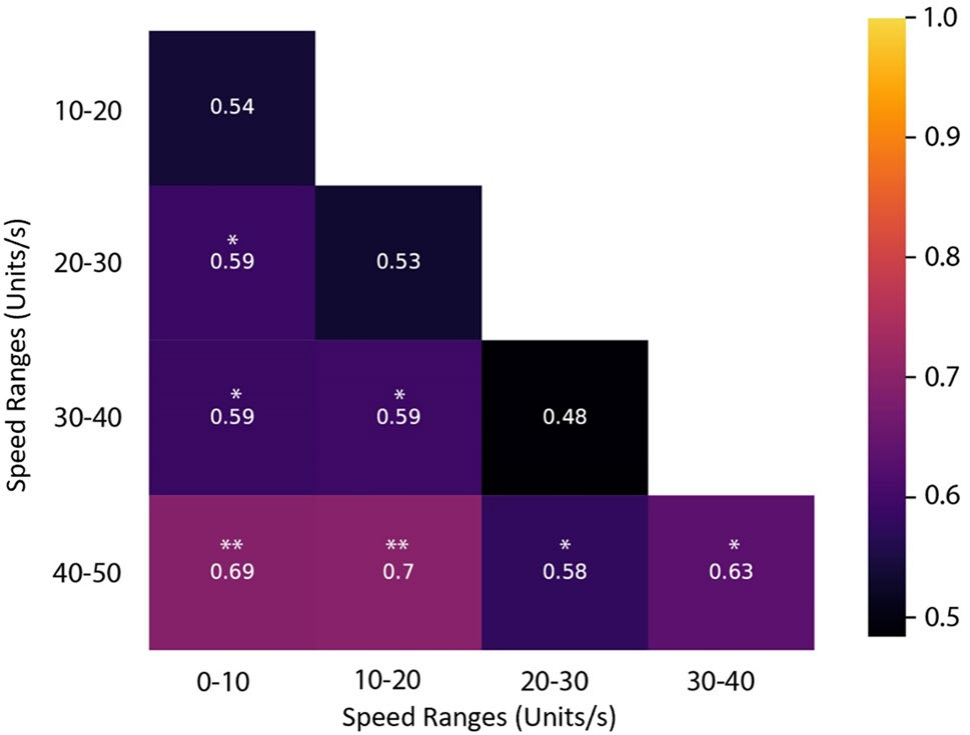
AUC’s for binary classifications of speed bins in patient one. Matrix showing classification results for binary classification between speed groups. Each square shows the AUC value resulting from the classification of the speeds labelled in the corresponding row and column. Significantly above chance AUC values marked with asterisk; multiple comparisons correction was performed with the maxT test.

We then analyzed how well the decoder could perform binary classifications between graded virtual-movement speeds for patient one. Seven out of ten classifications passed significance testing (Fig. 3). Further, we found a significant correlation between the speed difference between the classes compared and the decoding accuracy (r(10) = 0.79, *p* = 0.006), demonstrating that more separation between speed ranges lead to higher accuracy.

## Discussion

To the best of our knowledge, we are the first to demonstrate that movement speed can be decoded from human hippocampal theta utilizing a virtual-navigation task. Decoding the fastest vs slowest 10% of speeds resulted in significant decoding accuracy. These findings extend previous results showing that slow and fast movement speeds can be decoded during real-world ambulatory movement^26^. Further, we were able to classify between broad speed groups. These findings suggest that it’s possible to decode a range of speeds from hippocampal activity. Additionally, larger differences between the speed classes led to improved classification.

Unlike conventional EEG-based wheelchair designs that support only discrete control, our methodology offers a potential advantage by providing continuous decoding of movement speed. Decoding of discrete states may be insufficient for generating continuous, smoothly varying trajectories that can change at a moment’s notice. By contrast, our study aims to decode continuous movement speed in real-time from the hippocampus, a feature that has not been previously attempted in the context of BCI control. Incorporating decoded movement speed may aid in the development of effective and safe BCI wheelchair control methods.

Due to the naturalistic task design, our dataset is not perfectly balanced in terms of the time spent at various speeds. Furthermore, movement speed might be entangled with other factors during driving, such as turning intensity. While we aim to directly decode navigational behavior, high decoding accuracy can be achieved using secondary signals such as motor imagery or neurofeedback paradigm^4^. To improve performance, future research should explore how other features may improve virtual-movement speed decoding accuracy; for example, by including additional neural features and brain locations. To assess the feasibility of this method to be used in BCI it is important to assess which other higher-order navigational features, such as direction and self-location, can be decoded from human sEEG recordings as well as if such features can be decoded from imagined navigation.

These results represent the first step towards exploring the feasibility of using hippocampal signals, measured with intracranial depth electrodes, for a navigational neural prosthesis. This line of research may one day greatly benefit those suffering from paralysis, e.g. by enabling the development of a hippocampus-based cognitive BCI for wheelchair control.

## Methods

### Participants

**Figure 4:**
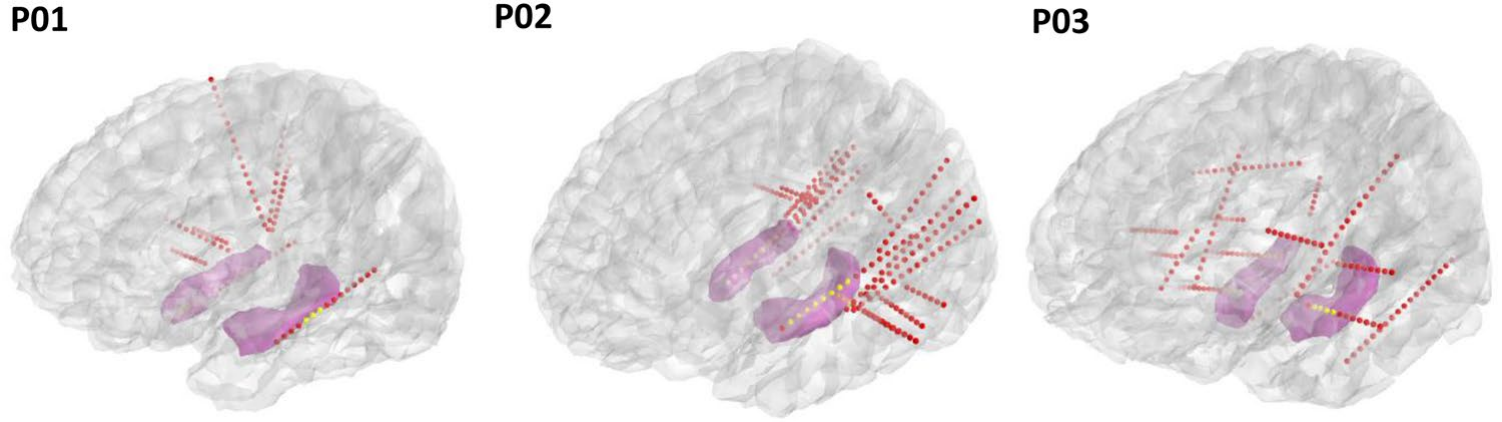
sEEG implantation locations. Electrodes inserted using stereotactically guided implantation. Electrode locations superimposed over the patient’s reconstructed brain from MRI.

Three participants (one female, two male, 24, 26, and 46 years of age) with pharmaco-resistant epilepsy were implanted with sEEG electrodes at the Maastricht University Medical Center (Fig. 4). These patients did not experience paralysis or significant motor impairments. The choice of participants with epilepsy in this study was to leverage the opportunity of sEEG recordings in a clinical setting as a proof-of-concept for decoding hippocampal activity related to navigation. This study is not intended to provide immediate benefits to the epilepsy patients but rather to lay the groundwork for potential future applications in patients with paralysis or motor impairments. Implantation locations were based solely on clinical evaluation and confirmed using post-implantation computed tomography.

Stereo-electroencephalography (sEEG) electrode implantation has been used to treat epilepsy, and the risks associated with the procedure have been systematically reviewed^27^. In cases of refractory epilepsy, the benefits of sEEG implantation for seizure control have been demonstrated to outweigh the associated risks. Similarly, we believe that for patients experiencing paralysis, the potential to regain the ability to move through the use of a hippocampal BCI could also outweigh the risks associated with the implantation procedure. It is important to note that the patients in our study received the sEEG implants for the primary purpose of treating their epilepsy, and not for the purpose of BCI control. The study was approved by the Medical Ethics Review Committee of Maastricht University Medical Center and the local commission of Epilepsy Center Kempenhaeghe. Patients provided informed consent in accordance with the principles of the Declaration of Helsinki. After electrode implantation, the patient was transferred to the epilepsy monitoring unit at Epilepsy Center Kempenhaeghe.

### Data acquisition

All patients were implanted with bilateral hippocampal electrodes. All electrodes were DIXI MicroDeep electrodes (DIXI Medical, France). Electrodes had a diameter of 0.8 mm, a contact length of 2 mm, and a 1.5 mm intercontact distance. A Micromed amplifier was used to record data (Micromed, Italy, 1024 Hz sampling rate). Contacts were referenced to a common white matter contact. Task and neural data were synced via LabStreamingLayer^28^.

Pre-operative three-tesla brain magnetic resonance imaging (MRI) and postoperative computed tomography (CT) were co-registered. An open-source Python package was then used to identify and label electrode locations within the hippocampus^29^. Participants had 11, 5, and 7 contacts within the right hippocampus and 3, 5, and 4 contacts in the left hippocampus, respectively.

### Procedure and task

The participants played a third-person delivery driver navigation video game on a laptop in their hospital room. A virtual environment was used because the patient was constrained to their hospital bed during the task. The game was custom-built using the Unity 3D game development platform (Unity Technologies, San Francisco, CA). The patient used the laptop keyboard, with the up/down keys controlling the speed of the car and the right/left keys controlling the turning direction, to drive through the three-dimensional virtual environment (Fig. 1a). Virtual movement speed refers to the rate at which the virtual vehicle traverses the environment. As the vehicle remains centered on the screen, the speed is determined by the movement of the surrounding environment, which provides a perception of displacement. While the forward key was pressed, the vehicle would accelerate at a steady rate before reaching maximum speed. Importantly, this means that there are no more key presses during fast speeds than during slow speeds. If the patient turned while driving too quickly, the car would tip over and reorient after a short delay, thus influencing the participant to regulate their speed. In addition to speed, the environment and task were designed to test whether it is possible to decode several features including direction, distance traveled, distance from boundaries, visual cues, self-location, path, and navigational planning.

The virtual environment consisted of a central loop with access to three visually distinct zones: a forest, city, and lake. The view of the three environmental zones was obstructed while on the central road to encourage participants to rely on their internal cognitive map, rather than simply responding to visual stimuli. First, the participant was presented with the keyboard controls and shown a top-down view of the environment (Fig. 1B). Next, participants were given unlimited time to freely explore to familiarize themselves with the environment and controls (172, 318, and 224 s, respectively). They were then presented with the task instructions. Subsequently, the primary task consisted of twelve trials each containing two phases: drop off and pick up (652, 559, and 1079 s, respectively). The car was reset to the starting position at the start of each phase (Fig. 1B, red ‘X’). During the drop-off phase, the patient was asked to drop off a package at one of three randomly selected zones. The package was automatically dropped off after either a short or long distance upon entering the target zone, regardless of the path they chose to take. Next, during the pick-up phase, the patient was instructed to navigate to the location of the package they dropped off in the preceding drop-off phase. Upon reaching the package, the patient was awarded points based on taking the optimal route and they were reset to the next drop-off phase.

### Data pre-processing

The linear trend was removed from the sEEG signals during a given session. To be used in decoding analyses, dynamic power in the theta (4–8 Hz) and gamma (52–99 Hz) frequencies were extracted. A Butterworth bandstop zero-phase (two-pass forward and reverse) non-causal filter and Hilbert transform were used to achieve continuous power signals.

These bands were chosen as the theta band is known to contain information about navigational processes and the gamma band closely resembles ensemble spiking^30^ which could also provide localized information about hippocampal processes. Spectral power and driving speed were windowed into one-second bins with a half-second overlap by calculating the mean of each bin. This way, each window of neural activity was assigned the corresponding speed value. Theta and gamma power were then log-transformed and z-scored across temporal windows.

### Decoding analysis

For each participant, a separate classifier was trained and tested, using a ten-fold cross-validation scheme performed within each participant's data. In this method, 9/10 of the data are used to fit the model and the remaining 1/10 is used for evaluation. This is repeated until each sample was used for testing exactly once. To classify movement speeds, linear discriminant analyses (LDA) were used^31^. LDA was chosen because of its resilience toward unbalanced data sets^32^. We used shrinkage regularization with the shrinkage parameter determined by the analytical procedure described in^33^.

Classifier performance was evaluated by plotting receiver operating characteristic (ROC) curves and calculating the corresponding area under the curve (AUC)^34^. To determine if classifier performance was above chance level, a bootstrapping resampling method was employed to estimate the null hypothesis distribution. In this method, labels were shifted 1000 times, and data were re-analyzed using the LDA; AUCs were then calculated for each replication. Shifting the data, rather than shuffling, was used to account for temporal autocorrelation between the neural and the speed data. AUCs that fell outside of the 95% confidence interval based on the null hypothesis were considered significant after correcting for multiple comparisons using the maxT test^35^).

To decode slow vs. fast virtual-movement speeds, neural signals were either labeled slow (slowest 10% of virtual-movement speeds) or fast (fastest 10% of virtual-movement speeds). Neural signals included theta and gamma power from the contacts located within the hippocampus. An LDA classifier was then trained to classify a range of virtual-movement speeds. For this analysis, speed data were discretized into five virtual-movement speed categories, each incorporating ten-speed units (e.g. 10–20 units/second, 20–30 units/second, etc.). We then performed ten binary classifications for each combination of the speed classes.

## Data Availability

Data available on request to the corresponding author.
